# Short-Term Outcomes of Intravitreal Faricimab Injection for Diabetic Macular Edema

**DOI:** 10.3390/medicina59040665

**Published:** 2023-03-27

**Authors:** Sentaro Kusuhara, Maya Kishimoto-Kishi, Wataru Matsumiya, Akiko Miki, Hisanori Imai, Makoto Nakamura

**Affiliations:** Division of Ophthalmology, Department of Surgery, Kobe University Graduate School of Medicine, 7-5-1 Kusunoki-cho, Chuo-ku, Kobe 650-0017, Japan

**Keywords:** diabetic macular edema, vascular endothelial growth factor, angiopoietin, faricimab, visual acuity, retinal thickness, safety

## Abstract

*Background and Objectives*: Faricimab is a novel bispecific antibody with Fab regions inhibiting both vascular endothelial growth factor-A and angiopoietin-2. Therefore, this study aimed to obtain short-term outcomes of intravitreal injection of faricimab (IVF) for the treatment of diabetic macular edema (DME) in daily clinical practice. *Materials and Methods*: A retrospective review was carried out on consecutive patients with DME who had been treated with IVF and were followed up for at least 1 month. Outcome measures included changes in logMAR best-corrected visual acuity (logMAR BCVA), central retinal thickness (CRT), number of IVF administrations, and safety. Clinical outcomes were also compared between the treatment-naïve and switch groups. *Results*: A total of 21 consecutive DME eyes from 19 patients were identified. The mean number of IVFs was 1.6 ± 0.8 during the mean follow-up time of 5.5 months. The overall mean logMAR BCVA following IVF was 0.236, 0.204, 0.190, and 0.224 at baseline, 1, 3, and 6 months, respectively, without a significant change from baseline to 1 month (*p* = 0.176) or for 6 months (*p* = 0.923). The overall mean CRT (μm) following IVF was 400.6, 346.6, 342.1, and 327.5 at baseline, 1, 3, and 6 months, respectively. CRT significantly decreased from baseline to 1 month (*p* = 0.001) but did not reach a significant level over 6 months following IVF (*p* = 0.070). No significant difference in BCVA or CRT was observed between the treatment-naïve and switch groups. No serious safety concerns were noted. *Conclusions*: IVF for the treatment of DME may preserve visual acuity and improve macular thickness without serious safety concerns in the short term in a real-world clinical setting.

## 1. Introduction

Previous basic studies clearly revealed that vascular endothelial growth factor-A (VEGF-A) and angiopoietin 2 (ANG2) are major players in the pathogenesis of diabetic macular edema (DME) [1]. However, the standard care of DME has long been continuous administration of anti-VEGF-A drugs via intravitreal injection [2,3]. The treatment strategy to keep intraocular VEGF-A levels below a certain level has been successful in many cases; however, the following issues were identified: approximately 20% of patients are refractory to anti-VEGF-A therapy (i.e., incomplete vision gain and/or persistent macular edema), and short treatment intervals are a burden for both patients and care providers [4]. Against the insufficient effectiveness background of anti-VEGF-A monotherapy, several attempts have been made in clinical trials to evaluate the efficacy and safety of combined blockade of VEGF-A and ANG2 signaling for DME [5,6,7].

Faricimab (Roche/Genentech, Basel, Switzerland) is a bispecific antibody with Fab regions binding to VEGF-A and ANG2 to act as both VEGF-A and ANG2 signal inhibitors. Based on the successful results of two multicenter phase 3 randomized clinical trials (RCTs) (YOSEMITE and RHINE), faricimab was approved and launched as a treatment for DME in 2022. YOSEMITE and RHINE trials demonstrated non-inferior vision gains and favorable anatomical outcomes in both faricimab every 8 weeks and personalized treatment interval (adjustable up to 16 weeks) arms as compared to aflibercept every 8 weeks arm at 1 year, indicating that patients with DME treated with faricimab might achieve optimal treatment outcomes even if the dosing interval is extended [7]. However, results of clinical trials in well-defined patient populations have been known to not necessarily guarantee effectiveness in diverse patient populations in real-world clinical settings [8]. For instance, the Protocol T extension study showed that visual gains achieved in RCTs were not maintained with subsequent standard care [9]. Accordingly, we collected and analyzed real-world data from patients with DME treated with intravitreal injection of faricimab (IVF) to obtain insights into the effectiveness and safety of faricimab in daily clinical practice.

## 2. Materials and Methods

### 2.1. Patients

We retrospectively reviewed the medical records of consecutive patients with DME who had been treated with IVF at Kobe University Hospital and followed up for at least 1 month. Approval for this study was granted by the institutional review board of the Kobe University Graduate School of Medicine (permission number: 170064). This study adhered to the tenets of the Declaration of Helsinki (7th revision) for research on human subjects, and the IRB waived obtaining informed consent from patients due to the retrospective nature of this study. However, patients were provided the opportunity to express their choice of data used by an opt-out system through the hospital’s website.

### 2.2. Data Collection

The following data were collected for analyses: age, sex, eye laterality, axial length, lens status, previous vitreous surgery, decimal best-corrected visual acuity (BCVA) (converted to the logarithm of the minimum angle of resolution [logMAR] for analyses), central retinal thickness (CRT) as determined by Macular Cube 200 × 200 scan data acquired by optical coherence tomography (Cirrus HD-OCT 5000, Carl Zeiss Meditec, Tokyo, Japan) (for one case who was not imaged by Cirrus HD-OCT 5000, the CRT value provided by Spectralis [Heidelberg Engineering, Heidelberg, Germany] was used), type of therapy (treatment-naïve or switch), the number of IVFs, type, and number of another treatment, follow-up period, and adverse events.

### 2.3. Intravitreal Faricimab Injection

Intravitreal faricimab injection (IVF) was conducted on an outpatient basis. Under ocular surface anesthesia with 4% lidocaine eyedrops, eyelid skin and ocular surface disinfection was performed using 5% povidone-iodine and 8-fold diluted PA IODO Ophthalmic and Eye washing Solution Disinfection (Nitten Pharmaceutical Co., Nagoya, Japan). An eyelid speculum was then placed to keep the eye open, and faricimab (6 mg/0.05 mL) (Roche/Genentech, Basel, Switzerland) was intravitreally injected using a 30-G needle (1/2 inch in length). Thereafter, antibiotic eyedrops and ointment were administered, the eyelid speculum was removed carefully, and an eyepatch was applied. The patient was instructed to remove the eyepatch the next day and continue antibiotic instillation (4 times daily) for 4 days.

### 2.4. Outcomes

The primary outcome was set as logMAR BCVA changes from baseline to 1 month after the first IVF, and secondary outcomes were logMAR BCVA changes at and after 2 months from baseline; CRT changes from baseline to each time point after the first IVF, and the number of IVFs, additional treatments, and complications/side effects during the follow-up period. The baseline value was defined as the nearest time point before the first IVF.

### 2.5. Statistical Analyses

Continuous variables were provided as mean ± standard deviation unless otherwise specified. Missing follow-up visit values were imputed using the last observation carried forward method for statistical analyses as needed. The generalized liner-mixed model was performed in the statistical analyses of changes among different time points using a graphical user interface for R software (The R Foundation for Statistical Computing, Vienna, Austria) and EZR (Saitama Medical Center, Jichi Medical University, Saitama, Japan). For other statistical analyses, MedCalc v.20.027 software (MedCalc Software, West-Vlaanderen, Belgium) was used. *p*-values of <0.05 were considered significant.

## 3. Results

### 3.1. Patients’ Characteristics

A total of 21 consecutive eyes from 19 patients were identified as fulfilling the inclusion criteria. The mean age was 67.7 ± 7.2 years; four patients (21%) were females, and five eyes (24%) had a history of prior vitrectomy. Among the 21 eyes, 14 (67%) were treatment-naïve before IVF, and the distribution of diabetic retinopathy severity was 19%, 71%, and 10% for moderate nonproliferative diabetic retinopathy (moderate NPDR), severe nonproliferative diabetic retinopathy (severe NPDR), and proliferative diabetic retinopathy (PDR), respectively. The mean follow-up time was 5.5 ± 2.0 months, and 1 case (5%) had a follow-up period of only 1 month. The summary of patients’ characteristics is shown in Table 1.

In the switch group, treatments performed within 6 months before the first IVF were intravitreal aflibercept (2 mg) injection (*n* = 7), intravitreal ranibizumab (0.5 mg) injection (*n* = 1), and macular photocoagulation (*n* = 1).

### 3.2. Treatment Outcomes

The scheme of the intravitreal injection was as needed (*pro re nata*: PRN). During the mean follow-up time of 5.5 months, the mean number of IVF was 1.6 ± 0.8 (median 1; range 1–3), and 12 cases (57%) had only 1 faricimab injection. The vitrectomized eyes had received more IVF than the non-vitrectomized eyes (0.6 ± 0.8 injections/month vs. 0.3 ± 0.3 injections/month, *p* = 0.042 [Mann-Whitney test]). Of 21 eyes, 12 cases (57%) had only one faricimab injection, and 15 eyes (71%) continued the IVF treatment; however, the remaining six eyes (29%) switched to other treatments: intravitreal aflibercept (2 mg) injection (*n* = 2), intravitreal brolucizumab (6 mg) injection (*n* = 2), sub-Tenon’s injection of triamcinolone acetonide (*n* = 2), and pars plana vitrectomy (*n* = 1). The treatments were switched because of insufficient CRT decrease in two eyes, CRT worsening in two eyes, and faricimab side effects in two eyes. Of six switched cases from IVF, four cases (67%) were treatment-naïve, and two cases (33%) were switched cases at baseline.

**Figure 1:**
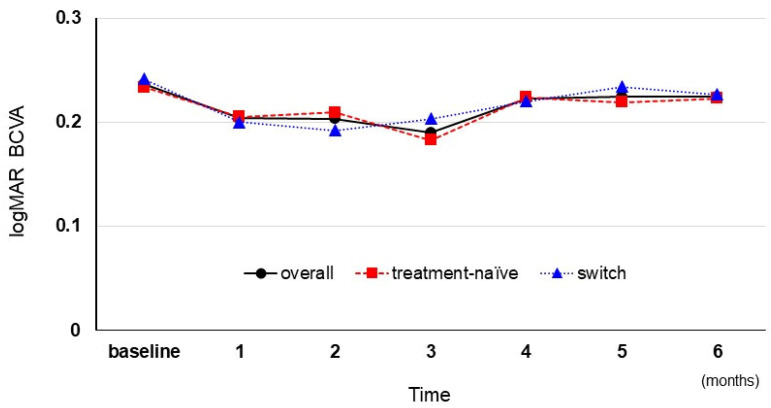
The mean logMAR BCVA changes following intravitreal faricimab injection. logMAR, the logarithm of the minimum angle of resolution; BCVA, best-corrected visual acuity.

The overall mean logMAR BCVA following IVF was 0.236 ± 0.242, 0.204 ± 0.257, 0.203 ± 0.254, 0.190 ± 0.277, 0.223 ± 0.291, 0.224 ± 0.301, and 0.224 ± 0.301 at baseline, 1, 2, 3, 4, 5, and 6 months, respectively. A significant logMAR BCVA change was not observed from baseline to 1 month (*p* = 0.176) or over 6 months following the IVF (*p* = 0.923). The mean logMAR BCVA in the treatment-naïve group was 0.234 ± 0.264, 0.205 ± 0.296, 0.209 ± 0.290, 0.183 ± 0.320, 0.224 ± 0.325, 0.219 ± 0.336, and 0.223 ± 0.324 at baseline, 1, 2, 3, 4, 5, and 6 months, respectively, whereas the mean logMAR BCVA in the switch group was 0.241 ± 0.209, 0.200 ± 0.175, 0.192 ± 0.178, 0.203 ± 0.183, 0.220 ± 0.230, 0.234 ± 0.238, and 0.227 ± 0.244 (Figure 1).

**Figure 2:**
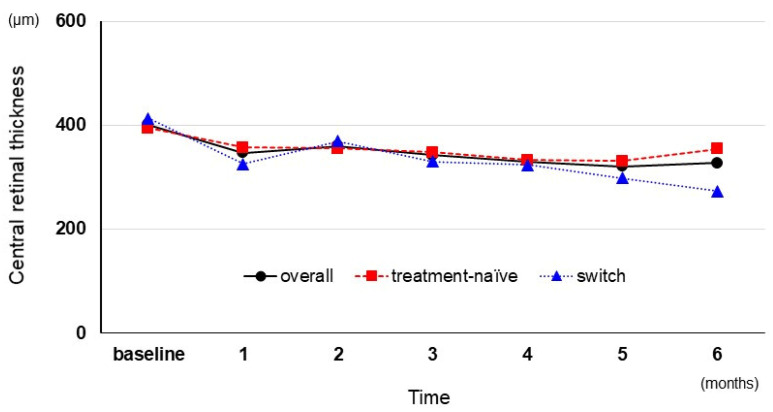
Changes in the mean central retinal thickness following the intravitreal faricimab injection.

Regarding the anatomical outcome, the overall mean CRT (μm) following the IVF was 400.6 ± 96.8, 346.6 ± 87.3, 359.8 ± 116.7, 342.1 ± 99.4, 329.8 ± 94.3, 320.3 ± 93.8, and 327.5 ± 99.4 at baseline, 1, 2, 3, 4, 5, and 6 months, respectively. CRT significantly decreased from baseline to 1 month (*p* = 0.001), although the CRT change did not reach a significant level over 6 months following the IVF (*p* = 0.070). The mean CRT (μm) in the treatment-naïve group was 394.2 ± 112.1, 357.4 ± 100.6, 355.1 ± 103.0, 348.1 ± 118.2, 333.1 ± 106.5, 333.1 ± 107.9, and 354.6 ± 107.2 at 6 months, whereas the CRT (μm) in the switch group was 413.4 ± 60.8, 325.0 ± 51.5, 369.1 ± 149.2, 330.3 ± 49.0, 323.1 ± 65.7, 298.1 ± 62.6, and 273.4 ± 54.3 at baseline, 1, 2, 3, 4, 5, and 6 months, respectively (Figure 2). The percentage of eyes that attained CRT of <325 μm was 52% at 1 month and 76% at the final visit.

**Figure 3:**
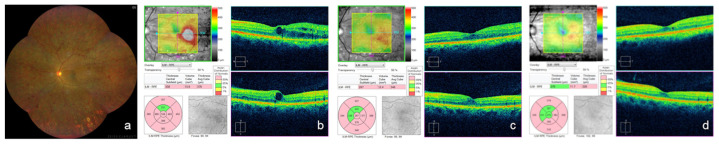
A treatment-naïve case (case 20) with good response to intravitreal faricimab injection. (**a**) a montage color fundus photograph at baseline; (**b**–**d**) optical coherence tomography images. Macular edema observed at baseline (**b**) disappeared 1 month after the intravitreal faricimab injection (**c**). No recurrence of macular edema was noted for up to 4 months without additional treatment.

**Figure 4:**
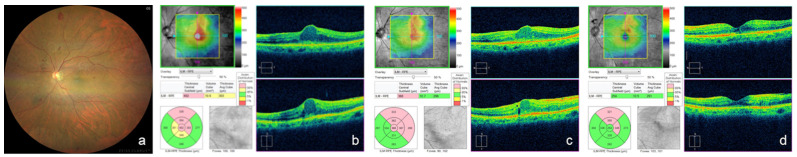
A switch case (case 14) with good response to the intravitreal faricimab injection. (**a**) A color fundus photograph at baseline; (**b**–**d**) optical coherence tomography images. Macular edema observed at baseline (**b**) slightly improved 1 month after the intravitreal faricimab injection (**c**) and almost disappeared at 4 months (**d**). No additional treatment was performed throughout the follow-up period.

**Figure 5:**
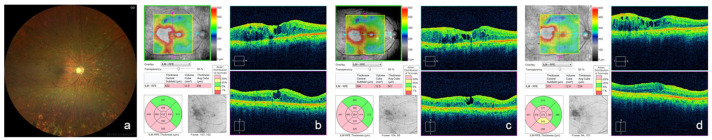
A treatment-naïve case (case 12) with a history of previous vitrectomy. (**a**) A color fundus photograph at baseline; (**b**–**d**) optical coherence tomography (OCT) images. Macular edema observed at baseline (**b**) remained unchanged during the intravitreal faricimab injection at 1 month (**c**). An OCT image at 5 months shows that the macular edema was refractory to the additional two intravitreal faricimab injections (**d**).

The detailed clinical courses of all patients are provided as Supplementary Material (Figure S1). The clinical courses of representative cases are shown in Figure 3, Figure 4 and Figure 5.

### 3.3. Safety

Although no intravitreal injection-related complications (e.g., bacterial endophthalmitis) were noted during the follow-up period, side effects in which a relationship to the administered drug (faricimab) cannot be denied were observed: worsening of macular edema (*n* = 2), epilepsy (*n* = 1), and anterior uveitis (*n* = 1). The eyes with macular edema deterioration were successfully treated with either intravitreal brolucizumab (6 mg) injection or pars plana vitrectomy. Epilepsy healed spontaneously, and anterior uveitis rapidly subsided with 0.1% fluorometholone eyedrops.

## 4. Discussion

Primary 1-year results from YOSEMITE and RHINE trials suggest that the IVF with dosing of up to every 16 weeks can potentially optimize real-world outcomes through its novel mechanism of dual VEGF-A and ANG2 pathway inhibition [7]. This retrospective study, which focused on the effectiveness and safety of faricimab in 21 DME eyes, aimed to add real-world evidence to existing CRT evidence.

Apart from YOSEMITE and RHINE trials, to the best of our knowledge, only one report retrospectively investigated the short-term outcomes of switching therapy from intravitreal aflibercept injection to IVF in treatment-resistant DME by Rush et al. [10]. Several differences were observed in baseline characteristics among YOSEMITE/RHINE trials (data for the personalized treatment interval group are presented hereafter), Rush’s study, and our study [7,10]. Although the mean age of 67.7 years in our study is comparable to that in YOSEMITE/RHINE trials (62.8/61.6 years) and Rush’s study (62.9 years), the proportion of female patients was smaller (21%) than in YOSEMITE/RHINE trials (37%/38%) and Rush’s study (50%). Because our study was conducted in Japan, all participants were Asians, whereas Whites made up the majority (77%/78%) of patients in YOSEMITE/RHINE trials. The mean HbA1c level was slightly higher in our study (8.4%) than that in YOSEMITE/RHINE trials (7.6%/7.7%) and Rush’s study (7.3%), and the severity of diabetic retinopathy was as follows: the percentage of eyes with severe NPDR or PDR was 81%, 74%/77%, and 38% in our study, YOSEMITE/RHINE trials, and Rush’s study, respectively. The mean logMAR BCVA of 0.24 in our study was better than that in YOSEMITE/RHINE trials (0.523/0.523 converted from letter score) and Rush’s study (0.60), and the mean CRT (μm) was poorer in YOSEMITE/RHINE trials (485.8/471.3) than that in our study (400.6) and Rush’s study (400.2). Treatment-naïve cases accounted for 67% of our study, whereas 78%/80% of cases were anti-VEGF treatment-naïve in YOSEMITE/RHINE trials (no treatment-naïve case was included in Rush’s study). Unexpectedly, no significant difference was observed in both visual and anatomical outcomes between the treatment-naïve and switch groups in our study. Therefore, we discuss the outcomes of our study using overall data.

YOSEMITE/RHINE trials demonstrated overt gains in the mean BCVA letters from 1 month after IVF [7]. A retrospective study of treatment-resistant DME by Rush et al. also found a significant improvement in the mean logMAR BCVA from baseline (0.60) to 4 months after IVF (0.50) [10]. However, the mean logMAR BCVA changes were not statistically significant throughout the follow-up period in our study. We do not suppose that BCVA results observed in our study denote an inadequate treatment effect of IVF because the mean absolute logMAR BCVA at 4 months (0.223) is better than that in Rush’s study (0.500). The ceiling effect can explain the difference in BCVA results among these four studies because the proportion of eyes with logMAR BCVA of ≤0.097 (Snellen BCVA, ≥20/25) was 33% at baseline and 43% at 1 month in our study. In addition, only one case showed a worsening visual acuity (increase in logMAR BCVA, ≥0.30). These findings suggest that good vision at baseline was maintained with IVF treatment. Accordingly, although further investigations are needed, the impact of IVF on visual outcomes seems satisfactory in daily clinical practice.

Anatomically, IVF significantly improved CRT in our study. The mean CRT decreased from 400.6 μm to 346.6 μm in 1 month. Of 21 eyes, 11 (52%) had a CRT of <325 μm at 1 month, and the proportion of eyes with CRT of <300 μm was 38% at 1 month and 71% at the final visit. CRT results in our study are non-inferior to those in YOSEMITE/RHINE trials and Rush’s study. In YOSEMITE/RHINE trials, approximately 40% of eyes attained complete disappearance of DME (defined as CRT <325 μm) at 1 month [7], and Rush et al. reported that 37.5% of eyes achieved a CRT of <300 μm at 4 months [10]. In comparing the mean CRT value at 4 months, our study (329.8 μm) likely shows a comparable anatomical status as in Rush’s study (340.3 μm), although a rescue treatment was performed in some cases in our study. Previous studies have shown superior anatomic improvement of faricimab in the management of DME compared to aflibercept. This is best explained by the idea that the pathogenesis of DME is related to ANG2 as well as VEGF-A [7,10,11]. The therapeutic approaches to persistent DME fall into two categories: stronger VEGF-A inhibition and simultaneous inhibition of VEGF-A and ANG2. Brolucizumab is a humanized monoclonal single-chain variable fragment that binds VEGF-A and acts as a potent anti-VEGF inhibitor. Phase 3 KITE & KESTREL trials showed a favorable reduction in central retinal thickness in the brolucizumab arm compared to the aflibercept arm [12]. However, the safety concerns of brolucizumab should be considered, which has become an issue in the treatment of age-related macular degeneration [13]. Therefore, faricimab might be an optimal treatment option, especially in persistent DME refractory to aflibercept.

Regarding the safety of IVF, we did not encounter any unexpected adverse events. Although a patient with epilepsy was examined after being hospitalized, the internist in charge believes that epilepsy was caused by systemic background. Accordingly, IVF for the treatment of DME is likely well tolerated in our study.

Most of the limitations of our study stem from the small number of cases which precluded us from performing detailed analyses on the true effects of treatment-naïve and switch groups, history of prior vitrectomy, treatment regimen on clinical outcomes, and safety. For example, our preliminary analysis showed that vitrectomized eyes tended to have more IVF than non-vitrectomized eyes. However, we do not know whether this can be generalized, as the number of vitrectomized eyes was too small. Although short-term outcomes of the IVF in the switch group help consider a direct contribution of ANG2 to DME pathology for each eye, the long-term effects of dual inhibition of VEGF-A and ANG2 in real-world clinical settings should be clarified in the future. We understand that the evidence level of this study is not high. However, given the paucity of real-world data on IVF for DME at the moment, we hope that short-term real-world data in this study will be of some help.

## 5. Conclusions

IVF for the treatment of DME may preserve visual acuity and improve macular thickness without serious safety concerns in the short term in a real-world clinical setting, which seems reasonable because both previous basic studies and recent clinical studies indicate the importance of VEGF-A and ANG2 in the pathogenesis and therapeutic targets of DME [1,5,6,7,10,14,15]. Further investigations are warranted to confirm the results of the current study.

## Figures and Tables

**Table 1:** Patients’ characteristics at baseline.

Characteristics	Data
Number of patients/eyes, *n*/*n*	19/21
Age (years), mean ± SD	67.7 ± 7.2
Sex, *n* (%)	
Male	15 (79)
Female	4 (21)
Eye, *n* (%)	
Right	10 (48)
Left	11 (52)
Axial length (mm), mean ± SD	24.1 ± 1.2
Previous intraocular surgery, *n* (%)	
Cataract surgery	11 (52)
Vitreous surgery	5 (24)
Best-corrected visual acuity (logMAR), mean ± SD	0.236 ± 0.242
Central retinal thickness (μm), mean ± SD	400.6 ± 96.8
HbA1c (%), mean ± SD	8.38 ± 2.50
Type of therapy, *n* (%)	
Treatment-naïve	14 (67)
Switch	7 (33)

Abbreviations: SD, standard deviation; logMAR, the logarithm of the minimum angle of resolution.

## Data Availability

Not applicable.

## Supplementary Materials

The following supporting information can be downloaded at https://www.mdpi.com/article/10.3390/medicina59040665/s1, Figure S1: The detailed clinical courses of all cases treated with intravitreal faricimab injection.
