# Supplementary material for: Short-Term Outcomes of Intravitreal Faricimab Injection for Diabetic Macular Edema
Source: Medicina (Kaunas). 2023 Mar 27;59(4):665. doi: 10.3390/medicina59040665 (PMC10142035; doi:10.3390/medicina59040665)
Supplement: Supplementary file 1 [file medicina-59-00665-s001.zip › medicina-2274975-supplementary.pdf]

Figure S1

The detailed clinical courses of all cases treated with intravitreal faricimab injection

Abbreviations

- BCVA: best-corrected visual acuity
- IVF: intravitreal faricimab injection
- IVA: intravitreal aflibercept injection
- IVBr: intravitreal brolucizumab injection
- STTA: sub-Tenon’s injection of triamcinolone acetonide
- PPV: pars plana vitrectomy

Case	Age range (years)	Sex	Type of therapy	Lens status	History of vitrectomy
Case 1	60s	male	Treatment-naïve	phakic	no

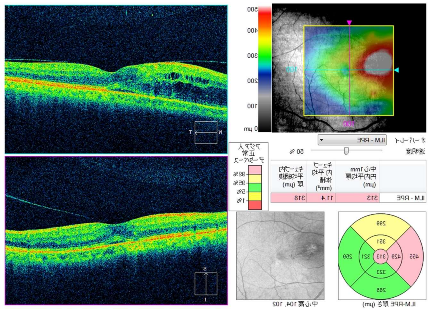

Baseline

BCVA	20/32
Treatment	IVF

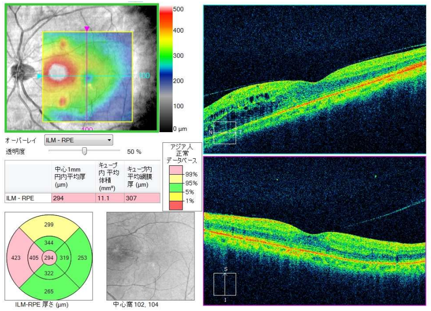

1 month

BCVA	-
Treatment	-

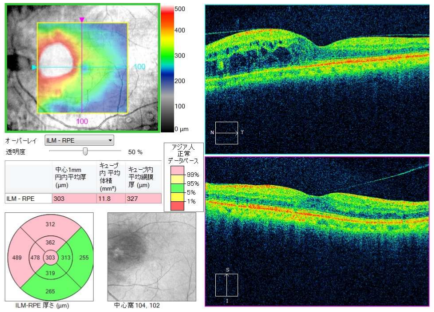

2 months

BCVA	20/40
Treatment	IVA

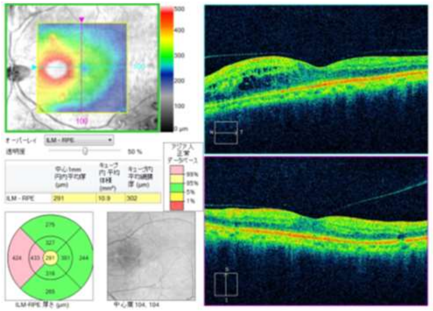

3 months

BCVA	20/40
Treatment	-

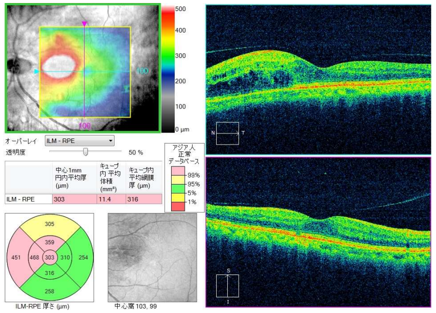

4 months

BCVA	-
Treatment	-

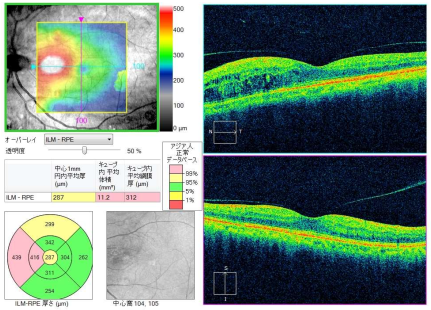

5 months

BCVA	20/40
Treatment	IVA

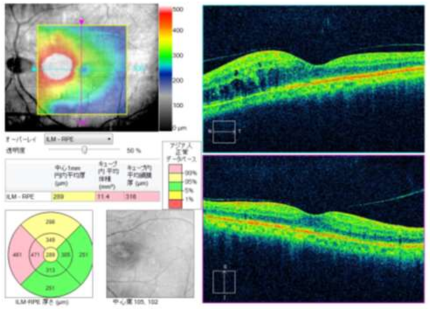

6 months

BCVA	20/40
Treatment	-

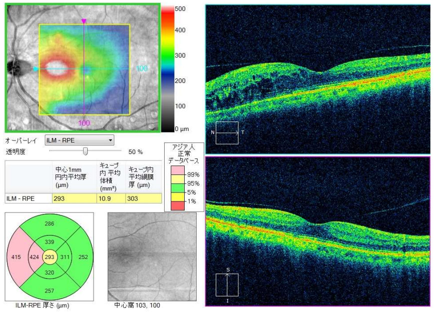

7 months

BCVA	20/50
Treatment	IVA

Case	Age range (years)	Sex	Type of therapy	Lens status	History of vitrectomy
Case 2	60s	male	Switch	IOL	no

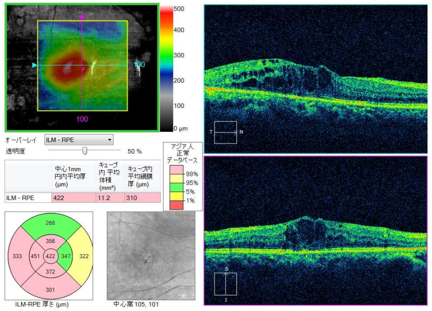

Baseline

BCVA	20/32
Treatment	IVF

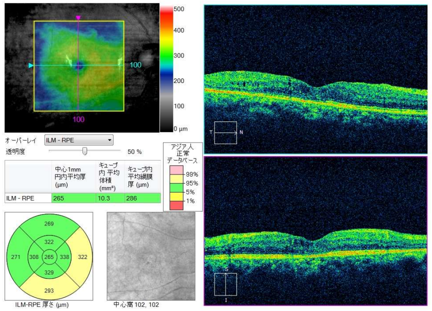

1 month

BCVA	20/32
Treatment	-

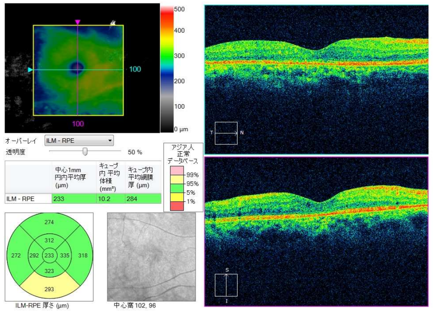

2 months

BCVA	20/25
Treatment	-

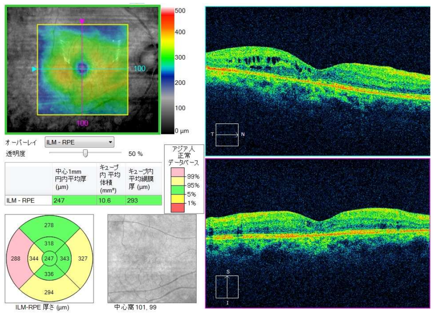

3 months

BCVA	20/25
Treatment	IVF

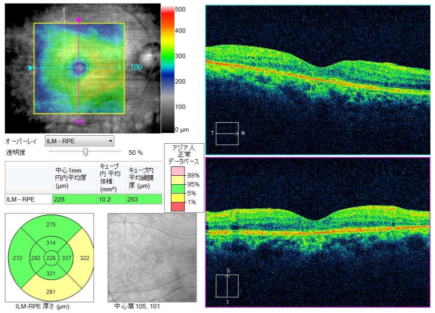

4 months

BCVA	20/25
Treatment	-

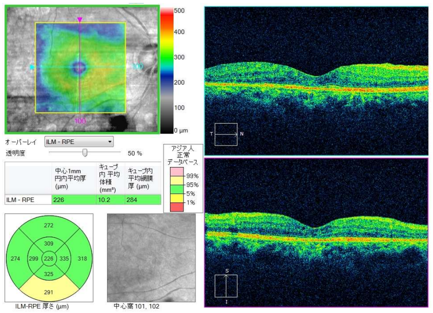

5 months

BCVA	-
Treatment	-

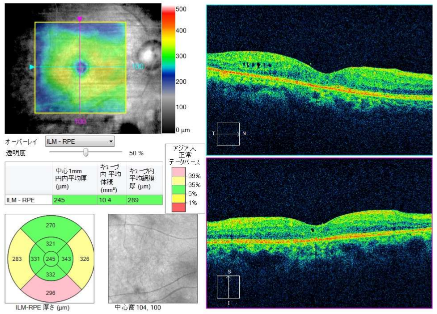

6 months

BCVA	20/25
Treatment	-

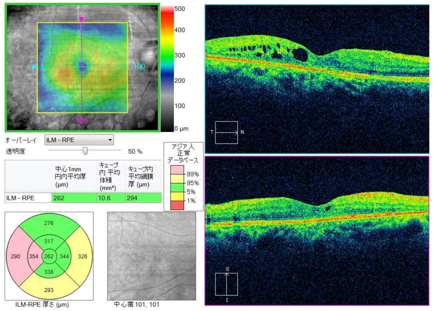

7 months

BCVA	20/25
Treatment	-

Case	Age range (years)	Sex	Type of therapy	Lens status	History of vitrectomy
Case 3	60s	male	Treatment-naïve	phakic	no

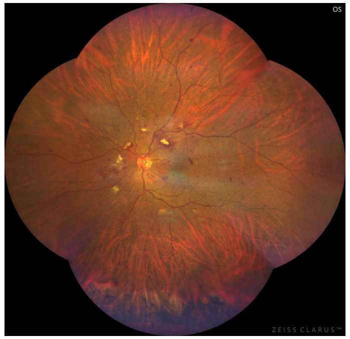

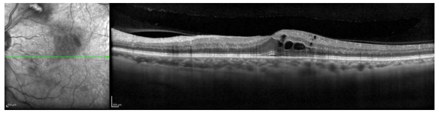

Baseline	
BCVA	20/32
Treatment	IVF

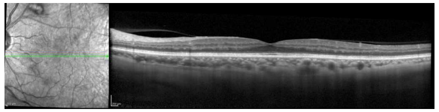

1 month	
BCVA	-
Treatment	-

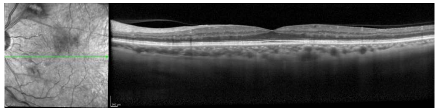

2 months	
BCVA	20/20
Treatment	-

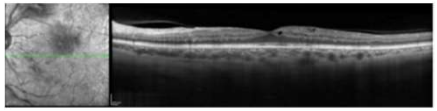

3 months	
BCVA	20/16
Treatment	IVF

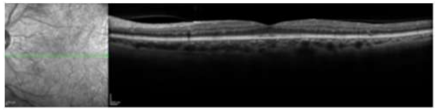

4 months	
BCVA	20/20
Treatment	-

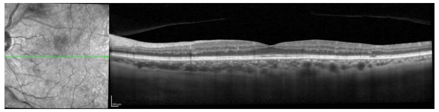

5 months	
BCVA	20/16
Treatment	-

6 months	
BCVA	-
Treatment	-

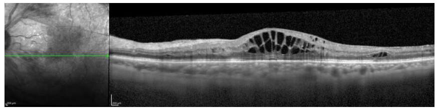

7 months	
BCVA	20/50
Treatment	IVF

Case	Age range (years)	Sex	Type of therapy	Lens status	History of vitrectomy
Case 4	60s	male	Treatment-naïve	phakic	no

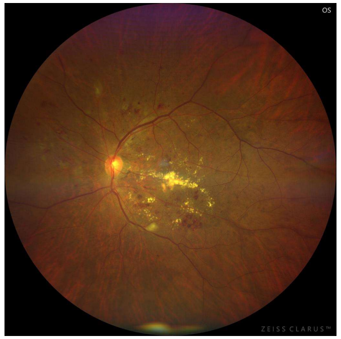

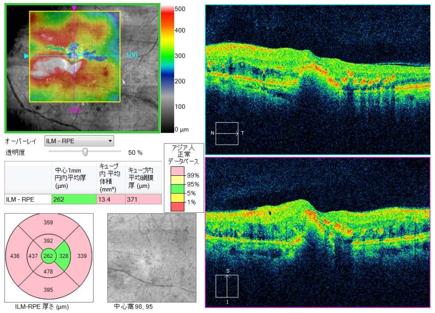

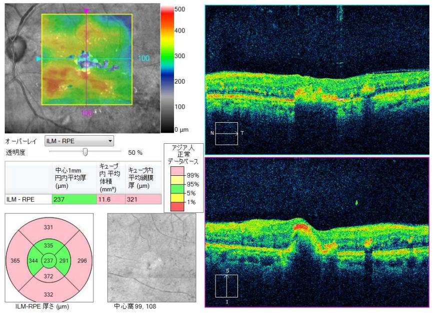

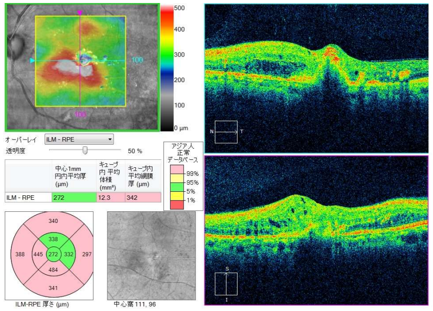

Baseline	
BCVA	20/160
Treatment	IVF

1 month	
BCVA	20/200
Treatment	-

2 months	
BCVA	-
Treatment	IVF

3 months	
BCVA	-
Treatment	-

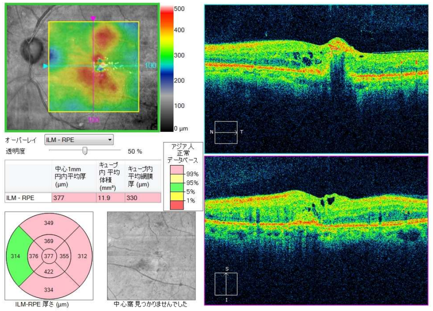

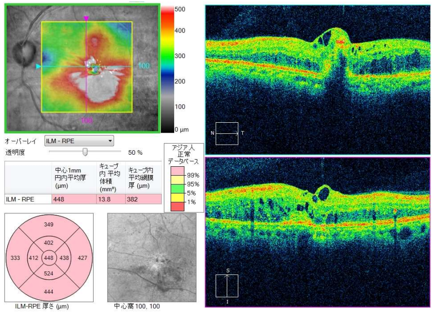

4 months	
BCVA	-
Treatment	-

5 months	
BCVA	-
Treatment	-

6 months	
BCVA	20/200
Treatment	-

7 months	
BCVA	20/200
Treatment	IVF

Case	Age range (years)	Sex	Type of therapy	Lens status	History of vitrectomy
Case 5	50s	male	Treatment-naïve	phakic	no

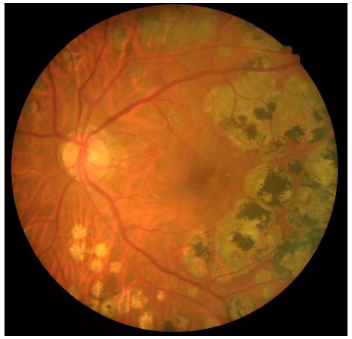

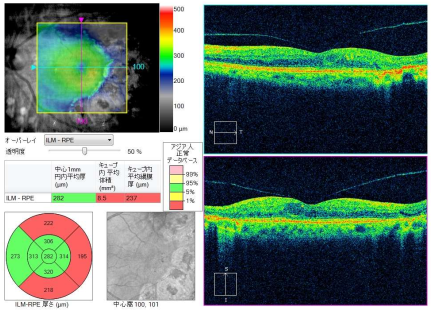

Baseline	
BCVA	20/20
Treatment	IVF

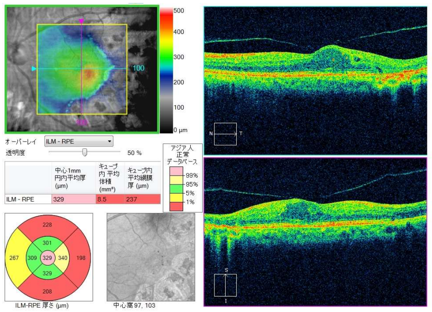

1 month	
BCVA	20/25
Treatment	IVBr

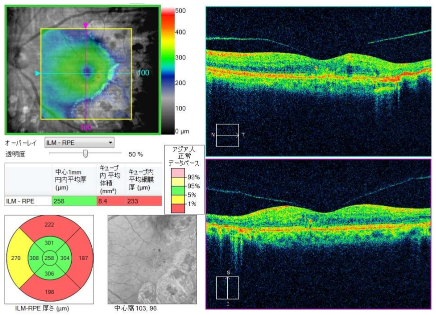

2 months	
BCVA	20/25
Treatment	-

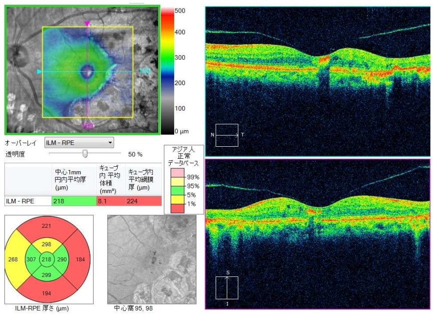

3 months	
BCVA	20/25
Treatment	-

4 months	
BCVA	-
Treatment	-

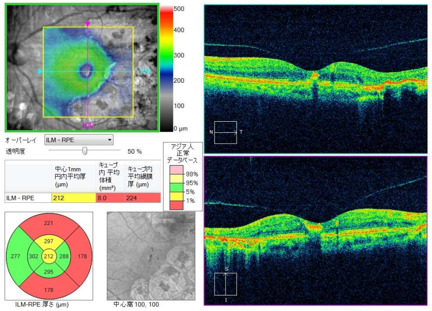

5 months	
BCVA	20/20
Treatment	-

6 months	
BCVA	-
Treatment	-

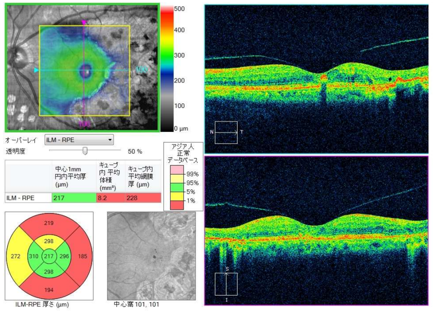

7 months	
BCVA	20/20
Treatment	-

Case	Age range (years)	Sex	Type of therapy	Lens status	History of vitrectomy
Case 6	60s	male	Treatment-naïve	phakic	no

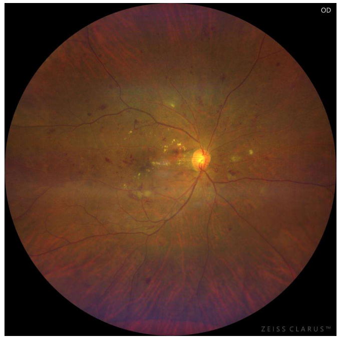

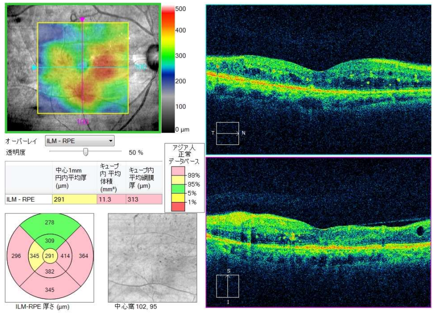

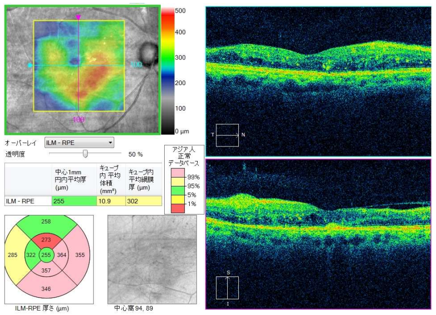

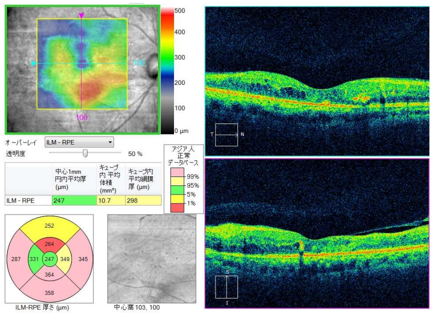

Baseline	
BCVA	20/80
Treatment	IVF

1 month	
BCVA	20/80
Treatment	-

2 months	
BCVA	-
Treatment	-

3 months	
BCVA	-
Treatment	-

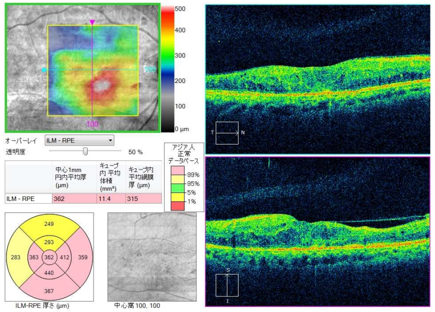

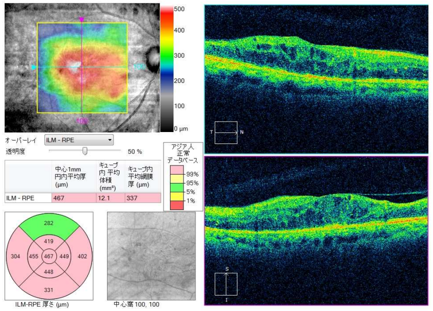

4 months	
BCVA	-
Treatment	-

5 months	
BCVA	-
Treatment	-

6 months	
BCVA	20/80
Treatment	-

7 months	
BCVA	20/80
Treatment	IVF

Case	Age range (years)	Sex	Type of therapy	Lens status	History of vitrectomy
Case 7	70s	male	Treatment-naïve	IOL	no

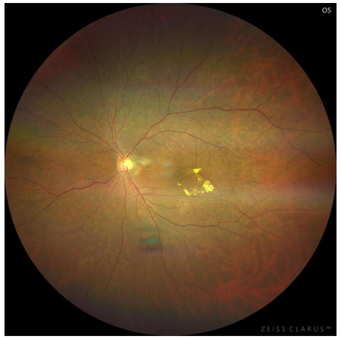

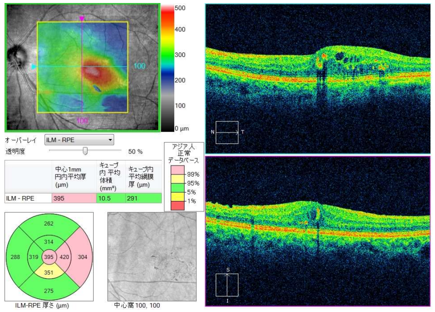

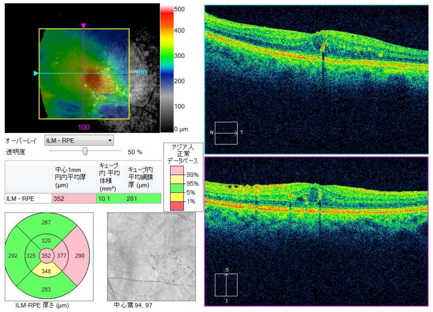

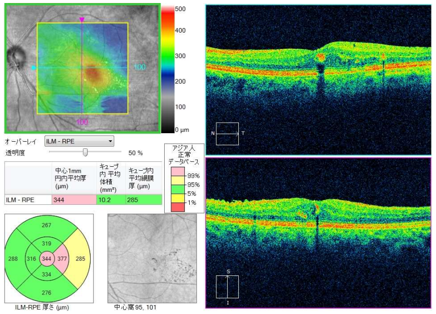

Baseline	
BCVA	20/20
Treatment	IVF

1 month	
BCVA	20/25
Treatment	-

2 months	
BCVA	20/20
Treatment	-

3 months	
BCVA	-
Treatment	-

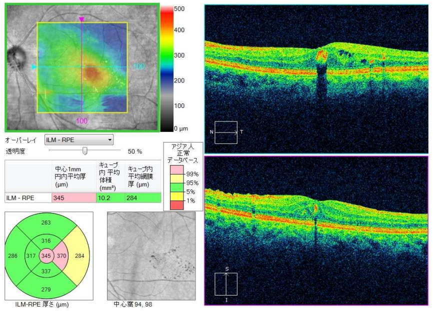

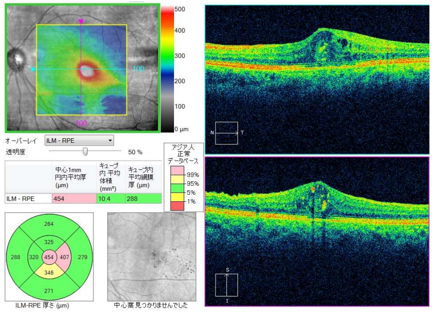

4 months	
BCVA	20/20
Treatment	-

5 months	
BCVA	-
Treatment	-

6 months	
BCVA	20/25
Treatment	-

Case	Age range (years)	Sex	Type of therapy	Lens status	History of vitrectomy
Case 8	60s	male	Treatment-naïve	phakic	no

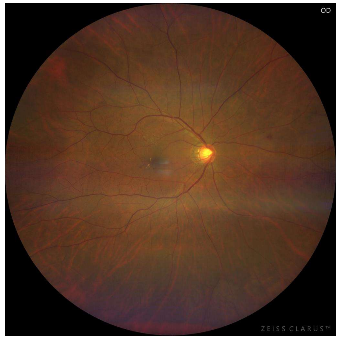

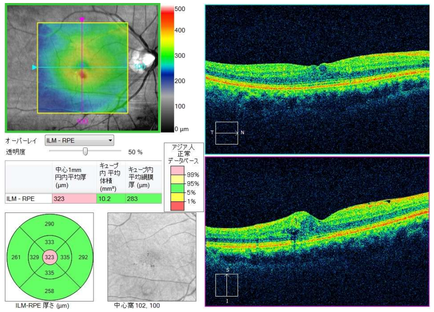

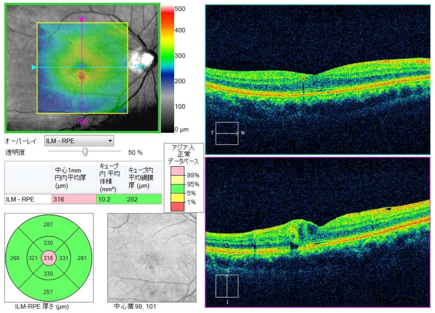

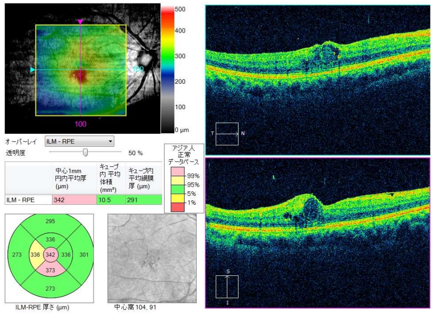

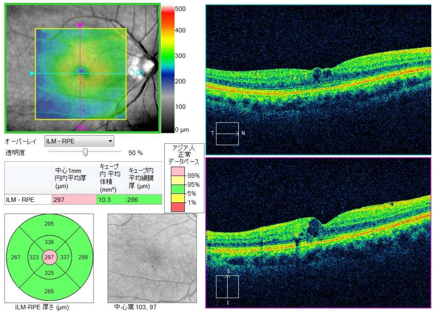

Baseline

BCVA	20/20
Treatment	IVF

1 month

BCVA	20/20
Treatment	IVBr

2 months

BCVA	20/40
Treatment	-

3 months

BCVA	20/20
Treatment	-

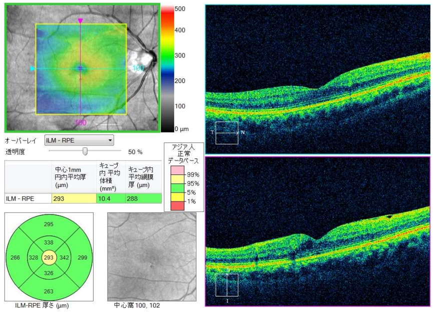

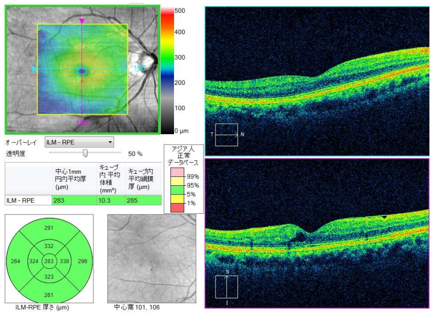

4 months

BCVA	20/20
Treatment	-

5 months

BCVA	-
Treatment	-

6 months

BCVA	-
Treatment	-

7 months

BCVA	20/20
Treatment	-

Case	Age range (years)	Sex	Type of therapy	Lens status	History of vitrectomy
Case 9	70s	male	Switch	IOL	no

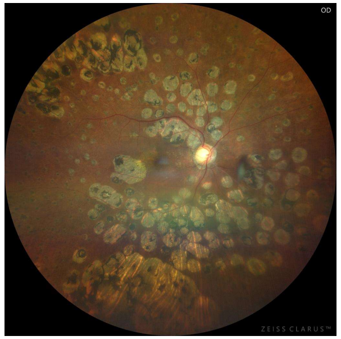

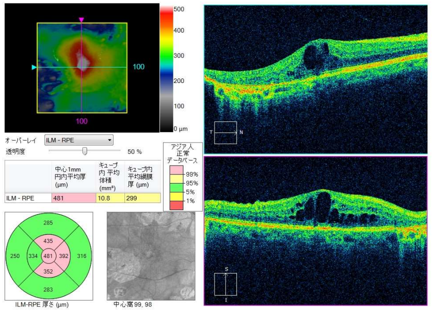

Baseline	
BCVA	20/80
Treatment	IVF

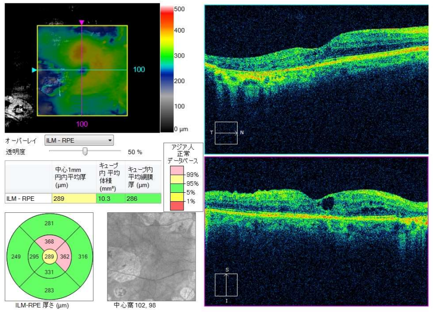

1 month	
BCVA	20/40
Treatment	-

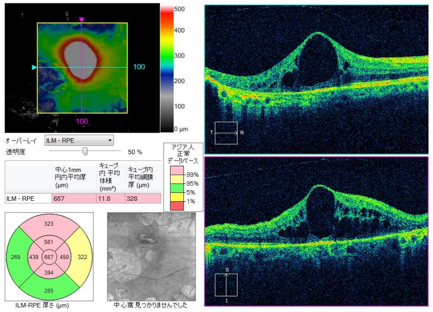

2 months	
BCVA	20/40
Treatment	PPV

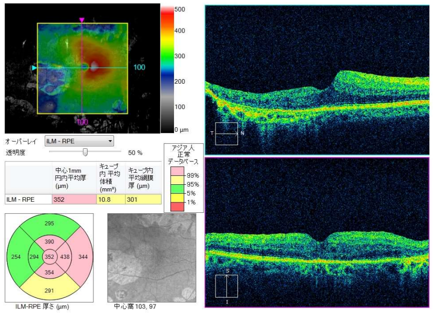

3 months	
BCVA	20/40
Treatment	-

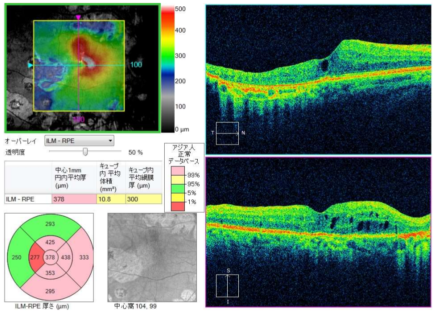

4 months	
BCVA	20/80
Treatment	-

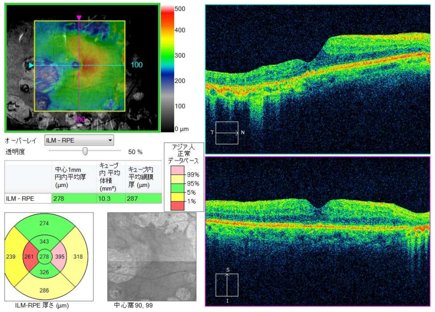

5 months	
BCVA	20/80
Treatment	-

Case	Age range (years)	Sex	Type of therapy	Lens status	History of vitrectomy
Case 10	60s	female	Treatment-naïve	IOL	yes

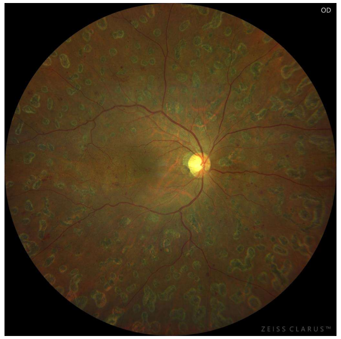

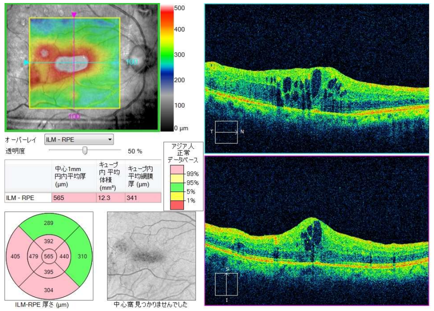

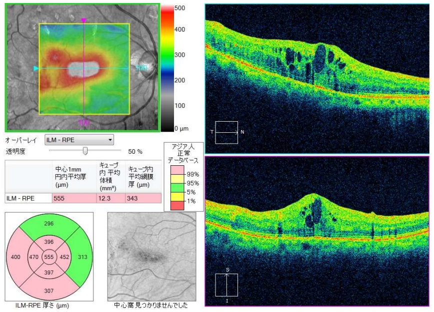

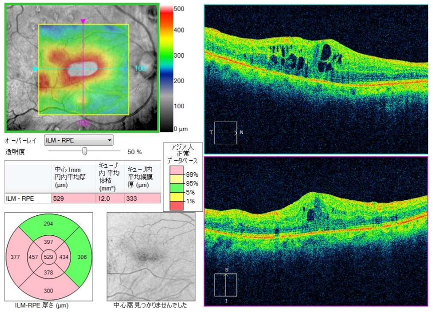

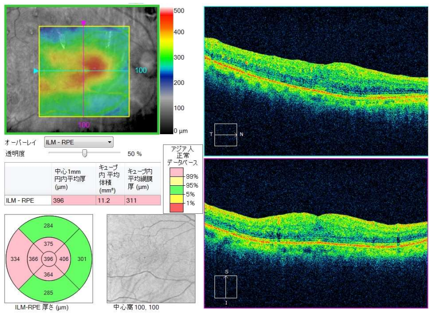

Baseline	
BCVA	20/100
Treatment	IVF

1 month	
BCVA	20/50
Treatment	IVF

2 months	
BCVA	-
Treatment	STTA

3 months	
BCVA	20/80
Treatment	-

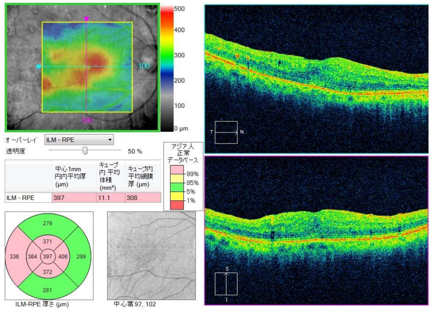

4 months	
BCVA	20/40
Treatment	-

5 months	
BCVA	20/40
Treatment	-

Case	Age range (years)	Sex	Type of therapy	Lens status	History of vitrectomy
Case 11	70s	female	Switch	IOL	no

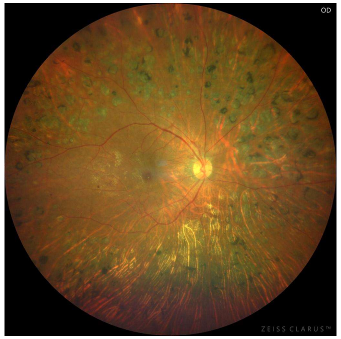

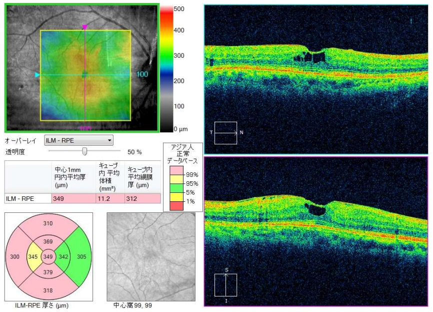

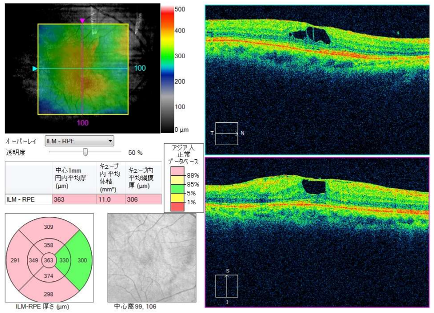

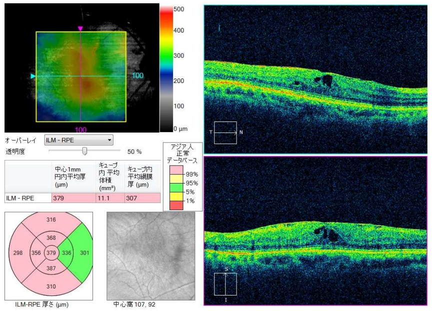

Baseline	
BCVA	20/25
Treatment	IVF

1 month	
BCVA	20/25
Treatment	-

2 months	
BCVA	20/25
Treatment	STTA

3 months	
BCVA	20/25
Treatment	PPV

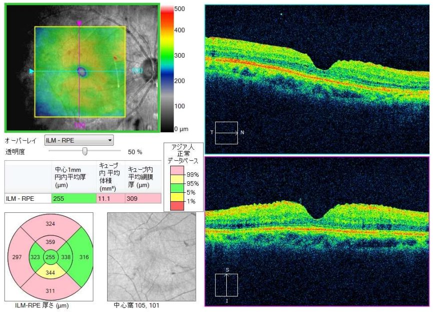

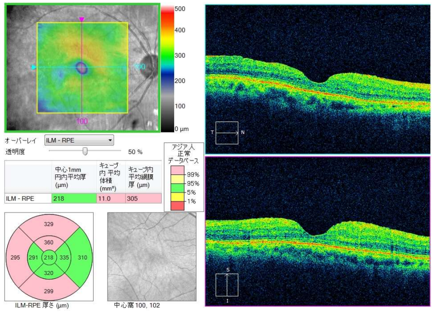

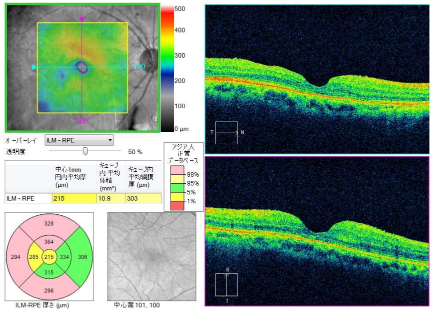

4 months	
BCVA	20/25
Treatment	-

5 months	
BCVA	-
Treatment	-

6 months	
BCVA	20/25
Treatment	STTA

7 months	
BCVA	20/25
Treatment	-

Case	Age range (years)	Sex	Type of therapy	Lens status	History of vitrectomy
Case 12	80s	male	Treatment-naïve	IOL	yes

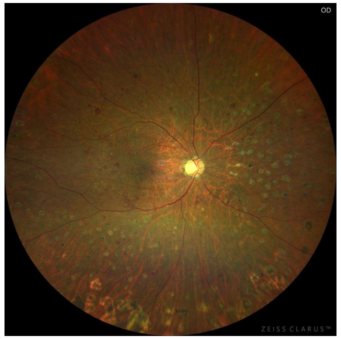

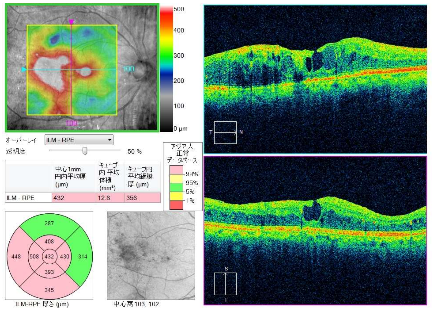

Baseline	
BCVA	20/40
Treatment	IVF

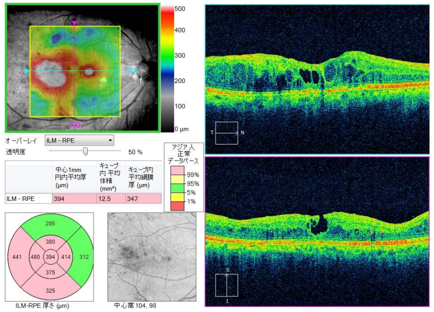

1 month	
BCVA	20/40
Treatment	IVF

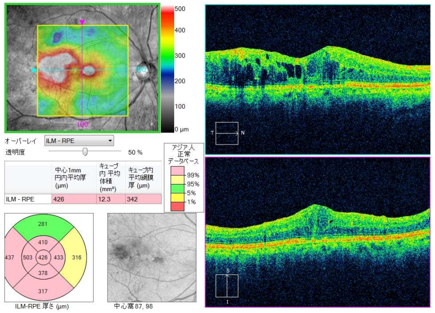

2 months	
BCVA	20/40
Treatment	IVF

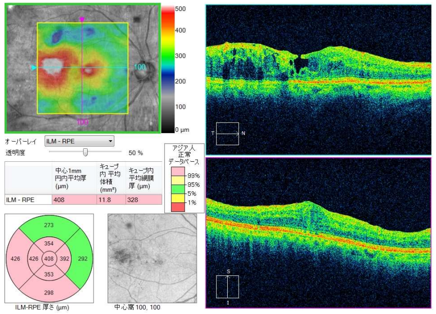

3 months	
BCVA	20/40
Treatment	-

4 months	
BCVA	-
Treatment	-

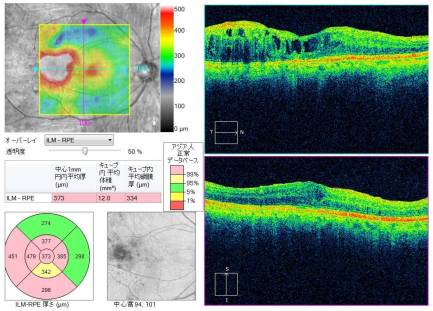

5 months	
BCVA	20/40
Treatment	-

Case	Age range (years)	Sex	Type of therapy	Lens status	History of vitrectomy
Case 13	70s	male	Switch	IOL	yes

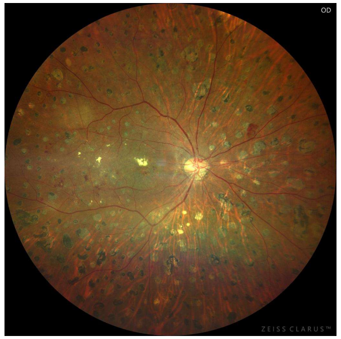

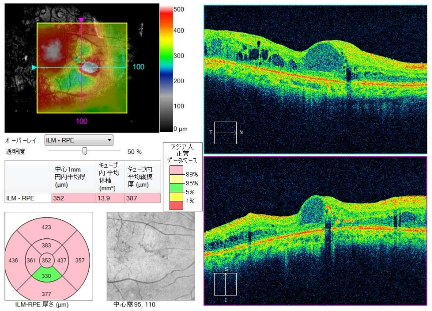

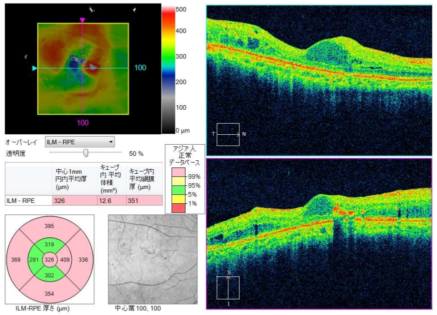

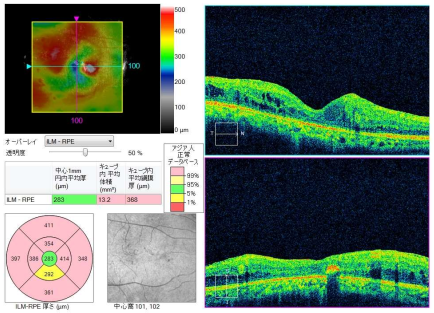

Baseline	
BCVA	20/40
Treatment	IVF

1 month	
BCVA	20/40
Treatment	-

2 months	
BCVA	-
Treatment	-

3 months	
BCVA	20/40
Treatment	-

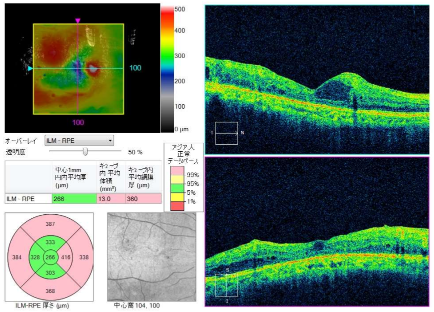

4 months	
BCVA	-
Treatment	-

5 months	
BCVA	20/50
Treatment	-

Case	Age range (years)	Sex	Type of therapy	Lens status	History of vitrectomy
Case 14	60s	female	Switch	IOL	no

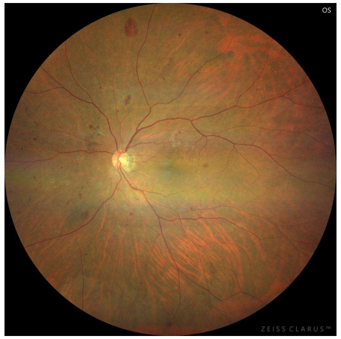

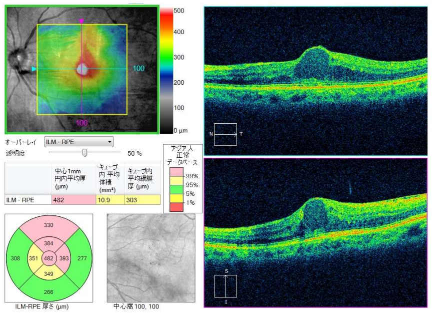

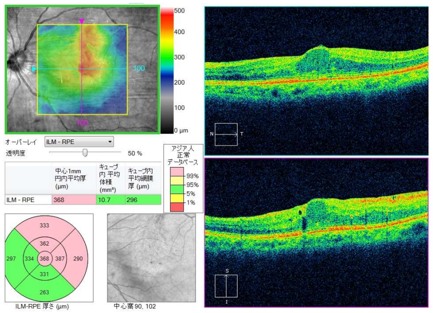

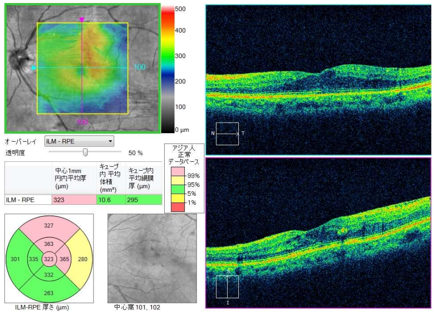

Baseline	
BCVA	20/40
Treatment	IVF

1 month	
BCVA	20/32
Treatment	-

2 months	
BCVA	20/32
Treatment	-

3 months	
BCVA	-
Treatment	-

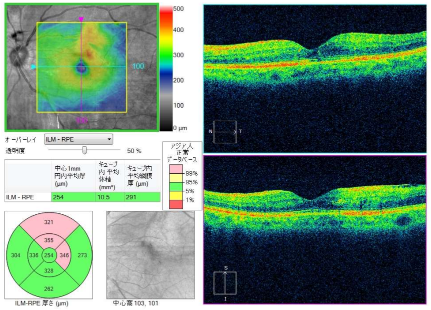

4 months	
BCVA	20/20
Treatment	-

Case	Age range (years)	Sex	Type of therapy	Lens status	History of vitrectomy
Case 15	60s	male	Treatment-naïve	phakic	no

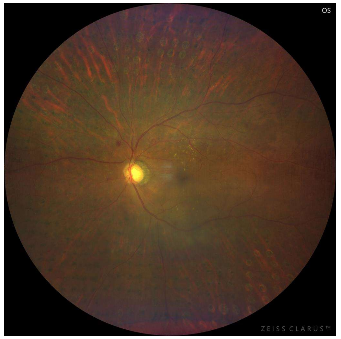

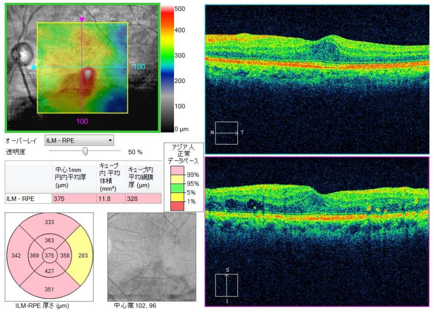

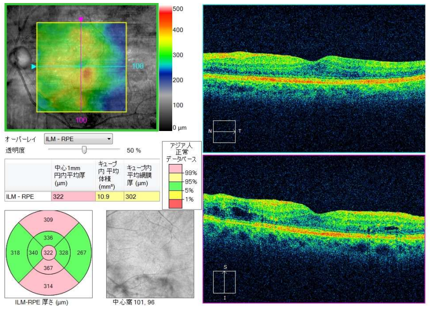

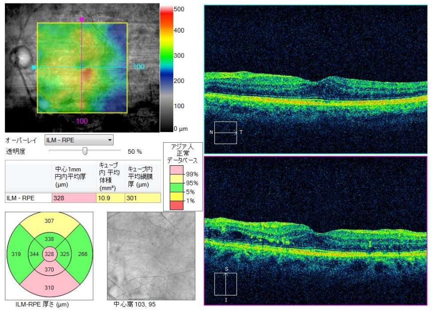

Baseline	
BCVA	20/25
Treatment	IVF

1 month	
BCVA	20/25
Treatment	-

2 months	
BCVA	20/20
Treatment	-

3 months	
BCVA	-
Treatment	-

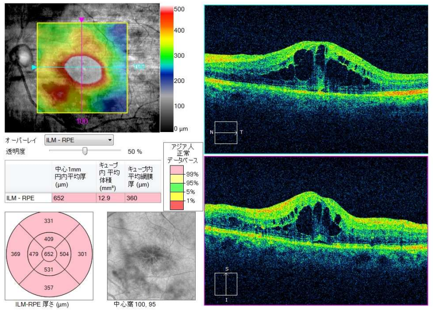

4 months	
BCVA	20/100
Treatment	-

Case	Age range (years)	Sex	Type of therapy	Lens status	History of vitrectomy
Case 16	60s	male	Treatment-naïve	phakic	no

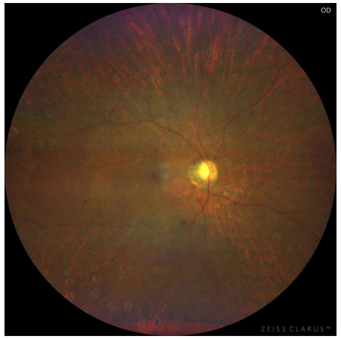

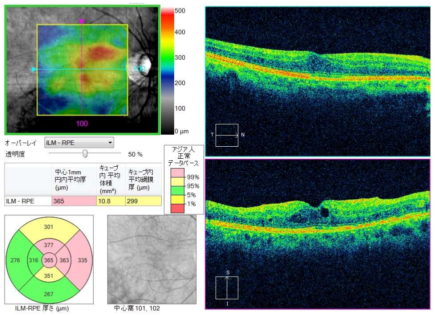

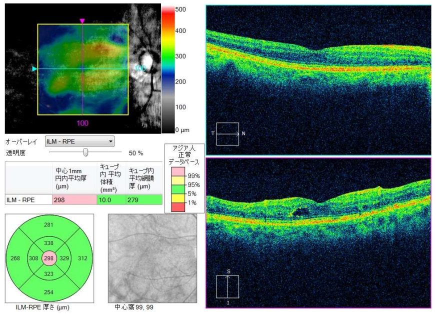

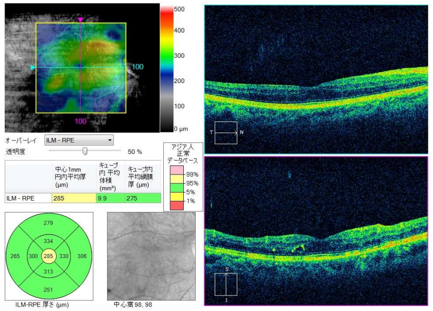

Baseline	
BCVA	20/40
Treatment	IVF

1 month	
BCVA	20/25
Treatment	-

2 months	
BCVA	20/20
Treatment	-

3 months	
BCVA	-
Treatment	-

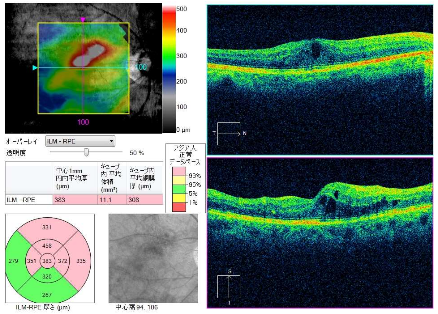

4 months	
BCVA	20/20
Treatment	IVF

Case	Age range (years)	Sex	Type of therapy	Lens status	History of vitrectomy
Case 17	70s	male	Treatment-naïve	phakic	no

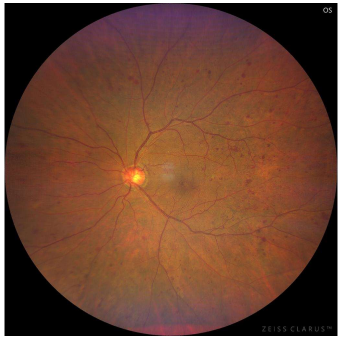

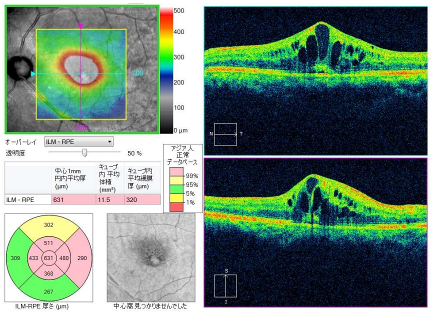

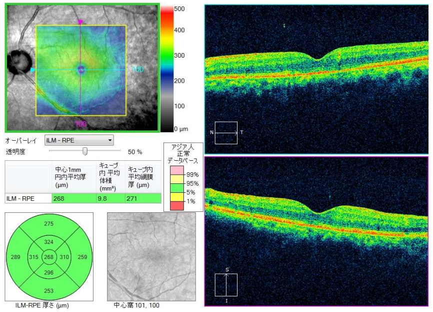

Baseline	
BCVA	20/20
Treatment	IVF

1 month	
BCVA	20/16
Treatment	-

2 months	
BCVA	-
Treatment	-

3 months	
BCVA	20/20
Treatment	-

Case	Age range (years)	Sex	Type of therapy	Lens status	History of vitrectomy
Case 18	50s	male	Switch	IOL	yes

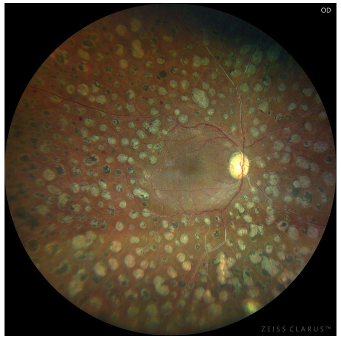

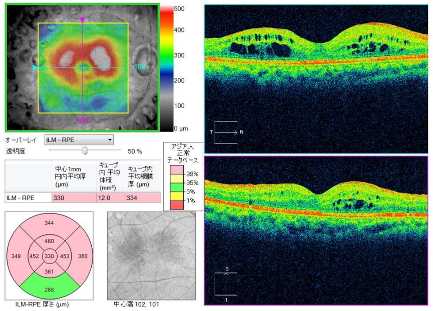

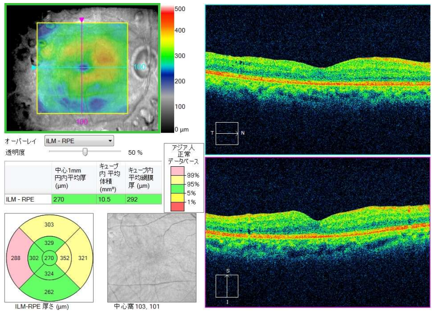

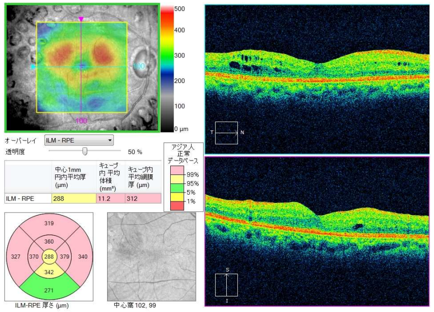

Baseline	
BCVA	20/20
Treatment	IVF

1 month	
BCVA	20/20
Treatment	-

2 months	
BCVA	-
Treatment	-

3 months	
BCVA	20/20
Treatment	IVF

Case	Age range (years)	Sex	Type of therapy	Lens status	History of vitrectomy
Case 19	70s	male	Switch	IOL	no

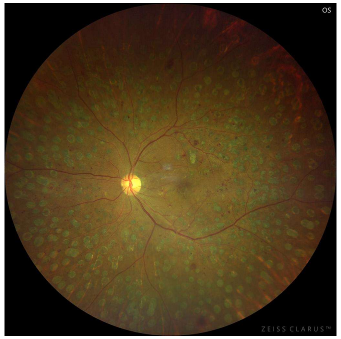

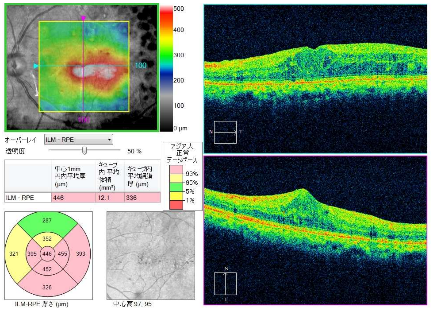

Baseline	
BCVA	20/80
Treatment	IVF

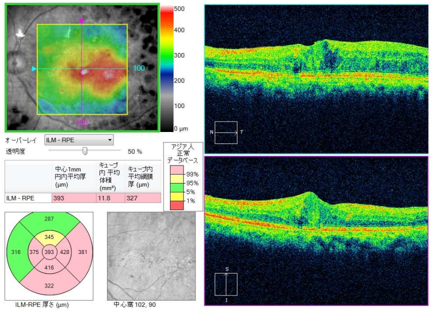

1 month	
BCVA	20/80
Treatment	IVF

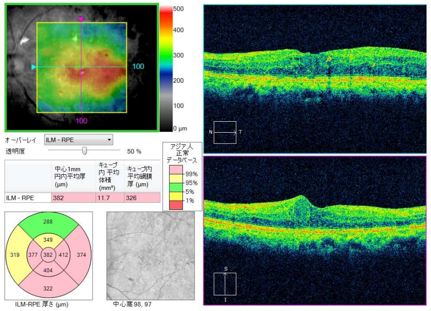

2 months	
BCVA	20/80
Treatment	IVF

3 months	
BCVA	-
Treatment	-

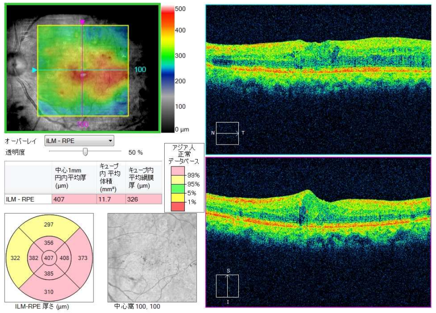

4 months	
BCVA	20/80
Treatment	-

Case	Age range (years)	Sex	Type of therapy	Lens status	History of vitrectomy
Case 20	50s	female	Treatment-naïve	phakic	no

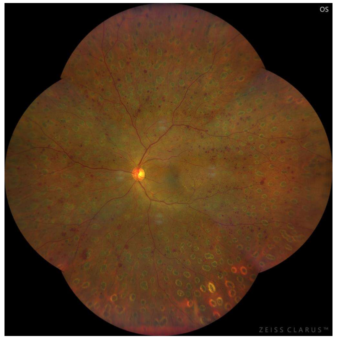

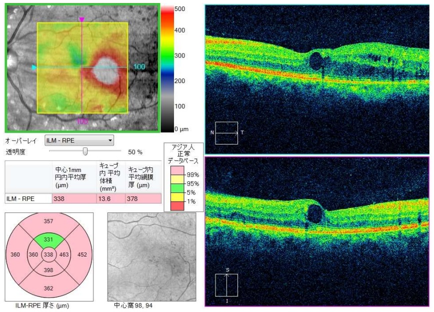

Baseline

BCVA	20/32
Treatment	IVF

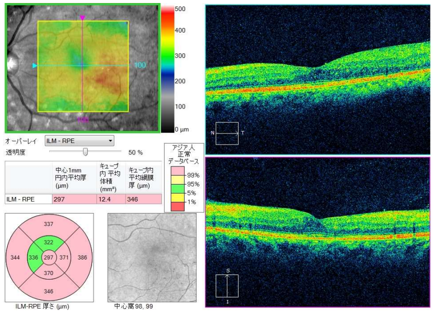

1 month

BCVA	20/25
Treatment	-

2 months

BCVA	-
Treatment	-

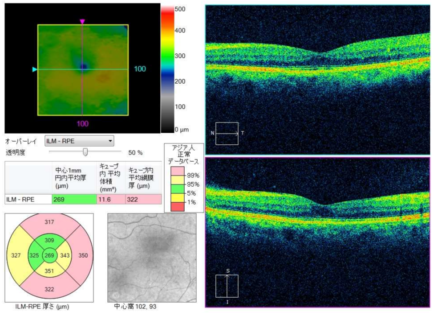

3 months

BCVA	20/20
Treatment	-

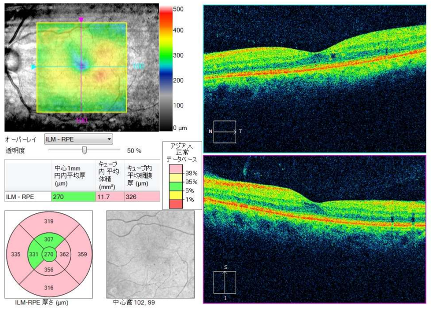

4 months

BCVA	20/20
Treatment	-

Case	Age range (years)	Sex	Type of therapy	Lens status	History of vitrectomy
Case 21	70s	male	Treatment-naïve	IOL	yes

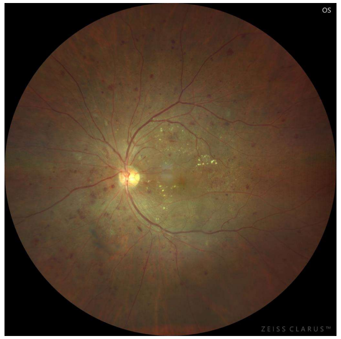

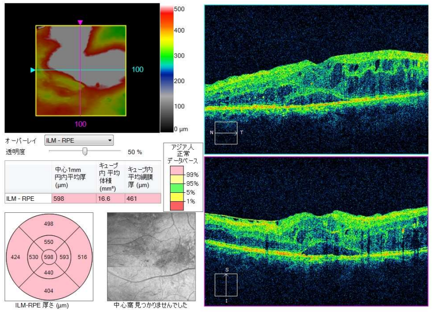

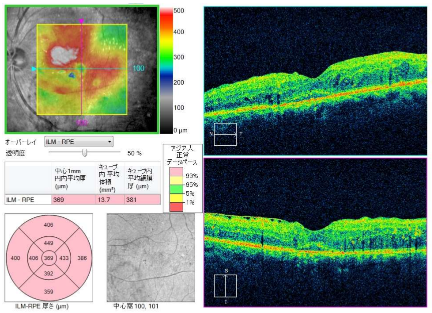

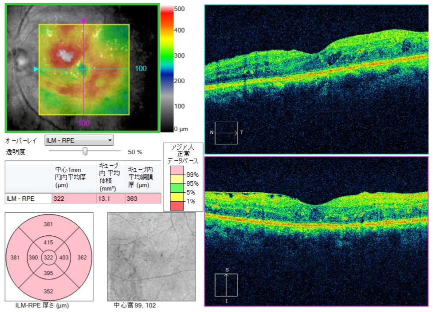

Baseline	
BCVA	20/40
Treatment	IVF

1 month	
BCVA	20/40
Treatment	-

2 months	
BCVA	20/32
Treatment	-
